# A Plasmid-Borne Gene Cluster Flanked by Two Restriction-Modification Systems Enables an Arctic Strain of *Psychrobacter* sp. to Decompose SDS

**DOI:** 10.3390/ijms25010551

**Published:** 2023-12-31

**Authors:** Robert Lasek, Ignacy Piszczek, Monika Krolikowski, Adrian Sówka, Dariusz Bartosik

**Affiliations:** Department of Bacterial Genetics, Institute of Microbiology, Faculty of Biology, University of Warsaw, Miecznikowa 1, 02-096 Warsaw, Poland; i.piszczek@student.uw.edu.pl (I.P.); monika.krolikowski@outlook.com (M.K.); a.sowka@nencki.edu.pl (A.S.)

**Keywords:** *Psychrobacter*, psychrophile, SDS degradation, alkyl sulfatase, restriction–modification system

## Abstract

The cold-adapted *Psychrobacter* sp. strain DAB_AL62B, isolated from ornithogenic deposits on the Arctic island of Spitsbergen, harbors a 34.5 kb plasmid, pP62BP1, which carries a genetic SLF module predicted to enable the host bacterium to metabolize alkyl sulfates including sodium dodecyl sulfate (SDS), a common anionic surfactant. In this work, we experimentally confirmed that the pP62BP1-harboring strain is capable of SDS degradation. The *slfCHSL* genes were shown to form an operon whose main promoter, P_slfC_, is negatively regulated by the product of the *slfR* gene in the absence of potential substrates. We showed that lauryl aldehyde acts as an inducer of the operon. The analysis of the draft genome sequence of the DAB_AL62B strain revealed that the crucial enzyme of the SDS degradation pathway—an alkyl sulfatase—is encoded only within the plasmid. The SLF module is flanked by two restriction–modification systems, which were shown to exhibit the same sequence specificity. We hypothesize that the maintenance of pP62BP1 may be dependent on this unique genetic organization.

## 1. Introduction

Microorganisms inhabiting cold environments have been extensively studied in past decades. Numerous psychrophilic or psychrotolerant bacteria have been isolated and subjected to genomic investigations. Among them, a large group includes bacteria of the genus *Psychrobacter* (class *Gammaproteobacteria*)—Gram-negative coccobacilli commonly found in permanently cold environments, including the Arctic and Antarctic regions.

The genus *Psychrobacter* was created in 1986 and originally contained only one species—*Psychrobacter immobilis*. Since then, numerous strains have been isolated and new species have been described. According to the NCBI Taxonomy Browser, the genus currently contains 48 species [1]. Psychrophilic strains of *Psychrobacter* spp. are of interest to many researchers, as they are commonly used as sources of cold-active enzymes (e.g., [2,3,4,5,6]) as well as models for the study of bacterial adaptations to low temperatures (e.g., [7,8,9]). However, knowledge about the natural plasmids harbored by *Psychrobacter* spp., which could be utilized as the basis for the construction of genetic tools to facilitate both basic and applied research in these bacteria, remains limited to only several functional studies [10,11,12,13,14,15,16].

**Figure 1:**
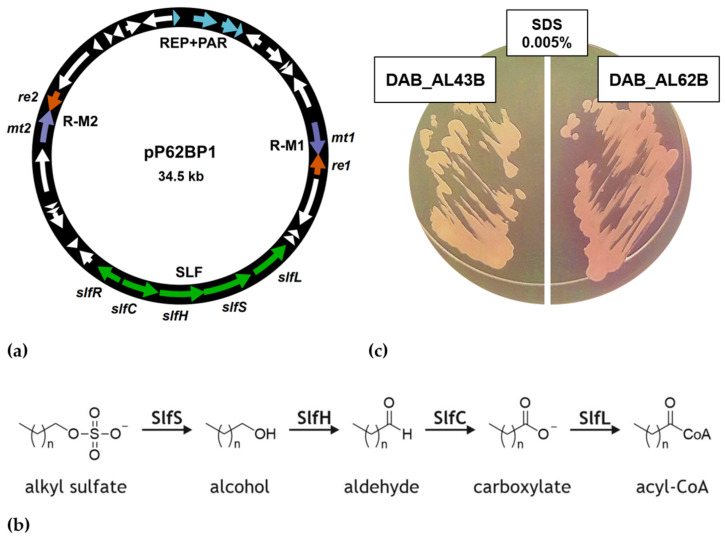
(**a**) Genetic organization of plasmid pP62BP1. Arrows indicate the open reading frames. Colors correspond to the main genetic modules of the plasmid: replication and partition module (REP + PAR); restriction–modification systems (R-M1, R-M2); and the SLF module. (**b**) Predicted metabolic pathway catalyzed by the products of genes identified within the SLF module (*slfCHSL*) [17]. (**c**) Growth of *Psychrobacter* sp. DAB_AL62B and DAB_AL43 (negative control) on LB medium supplemented with SDS to the final concentration of 0.005% (0.35 mM) and Stains-All dye. Zones of SDS degradation are visible as purple areas (image saturation was increased to 130% to emphasize the difference in color).

We previously reported on the in silico analysis of plasmid pP62BP1 of *Psychrobacter* sp. DAB_AL62B, a psychrotolerant strain isolated from guano sediments collected from a breeding colony of little auks (*Alle alle*) in the vicinity of the Polish Polar Station Hornsund (Spitsbergen, Svalbard Archipelago, Norway) (Figure 1a) [17]. One of the unique features of pP62BP1 is the presence of a compact gene cluster, named the SLF module, which was predicted to be involved in the metabolism of alkyl sulfates, e.g., sodium dodecyl sulfate (SDS), a common anionic surfactant [17]. The module consists of a gene for a transcriptional regulator of an AraC/XylS family (SlfR) and four tandemly oriented *slfCHSL* genes, which are predicted to encode: (i) an aldehyde oxidoreductase (SlfC), (ii) an alcohol dehydrogenase (SlfH), (iii) an alkyl sulfatase (SlfS), and (iv) a fatty-acid-CoA synthetase (SlfL) (Figure 1b) [17].

Curiously, it was observed that the SLF module was located within a 17 kb long fragment of pP62BP1 flanked by a pair of type II restriction–modification (R-M) systems that had a high level of mutual similarity. The amino acid sequences of putative methyltransferases (MTases) and restriction endonucleases (REases) that they encode were 99% and 96% identical, respectively. Based on comparative analyses, we predicted that the components of both R-M systems may target 5′-CCNGG-3′ pentamers [17].

In this work, we provide experimental confirmation of our initial predictions concerning the function of the SLF module and the sequence specificity of the ‘twin’ RM systems of pP62BP1. We also report the draft genome sequence of the pP62BP1 host strain, *Psychrobacter* sp. DAB_AL62B, which was obtained to explore the genetic context in which these unique plasmid-borne gene modules shape the phenotype of the host cell.

## 2. Results

### 2.1. Draft Genome Sequence of Psychrobacter sp. DAB_AL62B Reveals Its Close Similarity to Strains DAB_AL43B and DAB_AL32B

The draft genome sequence of *Psychrobacter* sp. DAB_AL62B consists of 3,215,484 bp with a GC content of 41.97%. Annotation with the NCBI Prokaryotic Genome Annotation Pipeline [18] resulted in the prediction of 2672 genes, including 2575 protein-coding genes, 52 RNA genes, and 45 pseudogenes. Of these, 31 genes were identified in contigs 17, 18, 24, and 34, which correspond to the previously obtained nucleotide sequence of plasmid pP62BP1 [17]. Following the minimal standards proposed by Chun et al. [19], the draft genome sequence was checked for completeness and contamination using a variant of the CheckM algorithm [20] implemented by the EvalG tool included in the PATRIC database annotation pipeline [21]. The analysis indicated a high level of completeness (99.6%) and low level of contamination (0.6%) for the assembly.

We used the Type Strain Genome Server (TYGS) [22] to investigate the relationship between the studied strain and 32 representative members of the genus *Psychrobacter* with the known genome sequence (Table S1), including strains DAB_AL43B and DAB_AL32B, isolated from the same ornithogenic deposits as DAB_AL62B [15]. The phylogenetic analysis of 16S rDNA sequences from this set of strains (Figure S1) showed that *Psychrobacter* sp. DAB_AL62B, although closely related, is grouped separately from strains DAB_AL43B and DAB_AL32B, as previously reported [15]. However, in the TYGS-generated phylogenomic tree of *Psychrobacter* species based on whole-genome distances, the three strains formed a monophyletic group, although they were not classified into the same species cluster (Figure S2). These results were corroborated by the analysis performed using the AAI-profiler web server (Figure S3, Table S2) [23]. Among all target species in the Uniprot database, the predicted proteomes of strains DAB_AL43B and DAB_AL32B showed the highest average amino acid identity to DAB_AL62B, at 0.969 and 0.971, respectively. Taxonomic profile analysis revealed that the proteins encoded in the genomes of these two strains were the closest matches for the DAB_AL62B proteins, at ca. 46% and ca. 43%, respectively. Similarly, the average nucleotide identity (ANI) and digital DNA–DNA hybridization (dDDH) values for the three genomes (within the ranges of 95.84–95.93% and 65.0–65.5, respectively) indicated their high level of relatedness (Figure S4).

Despite the observed similarity at the genomic level, the three closely related *Psychrobacter* strains differ in their plasmidome. While pP62BP1 is the only plasmid of strain DAB_AL62B, strain DAB_AL32B carries two plasmids, pP32BP1 (4.6 kb) and pP32BP2 (54.4 kb), whereas four plasmids were identified in strain DAB_AL43B (pP43BP1-4, 4.4–6.4 kb) [15]. Notable similarity was found between the replication and partition modules of plasmids pP62BP1 and pP32BP2 [12]; however, apart from this, the two plasmids do not share any homologous genes.

At the level of the predicted proteomes, the analysis of orthologous gene clusters by the OrthoVenn2 web server [24] showed that the three sister strains share 2244 gene clusters (Figure S5). Both the number of strain-specific clusters and the number of singletons (not assigned to any cluster) were lowest for DAB_AL62B (8 and 172, respectively; Figure S5). The COG (clusters of orthologous groups) functional analysis of protein sequences predicted to be encoded in the DAB_AL62B genome revealed that 955 proteins were assigned to categories encompassing cellular metabolism, including energy production and conversion (COG category C), as well as the transport and metabolism of carbohydrates (G), nucleotides (F), lipids (I), coenzymes (H), inorganic ions (P), and secondary metabolites (Q). The most abundant category, represented by 198 proteins, was amino acid transport and metabolism (COG category E), as previously observed for strain DAB_AL43B (Table S3) [13].

### 2.2. Psychrobacter sp. DAB_AL62B Is Capable of Alkyl Sulfate Degradation

The analysis of the DAB_AL62B genome content confirmed that there is no chromosomally encoded gene for an alkyl sulfatase, the crucial enzyme in the alkyl sulfate degradation pathway. This observation suggested that the predicted metabolic activity of *Psychrobacter* sp. DAB_AL62B could rely solely on the presence of the SLF module carried by pP62BP1.

To verify the predictions based on in silico analyses, we checked the ability of the studied strain to decompose SDS and its analogs. In all experiments, *Psychrobacter* sp. DAB_AL43B, which lacks any identifiable gene for an alkyl sulfatase, was used as a negative control. Our preliminary study revealed that the pP62BP1-harboring strain cannot use alkyl sulfates as the sole carbon and energy sources in a minimal medium. Consequently, we monitored the decomposition of alkyl sulfates in a rich solid medium (LB) supplemented with Stains-All dye based on the principle described by Rusconi et al. [25]. In this assay, *Psychrobacter* sp. DAB_AL62B was found to be able to degrade sodium dodecyl sulfate (Figure 1c) as well as its homologs with 8, 10, and 16 carbon atoms in their alkyl chains.

**Figure 2:**
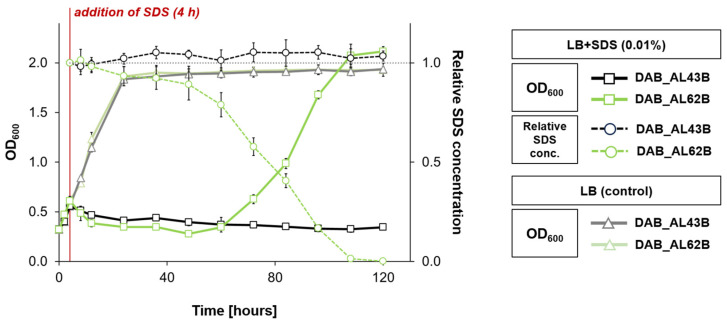
Growth of *Psychrobacter* sp. DAB_AL62B (green lines) and DAB_AL43B (negative control; black/grey lines) in the liquid medium supplemented with SDS to the final concentration of 0.01%. The bacterial cultures were preincubated for 4 h at 22 °C. Then, the cultures were divided into two flasks each, and SDS was added to one of the flasks, in which the OD_600_ was subsequently monitored (solid lines, squares). The flasks without SDS supplementation served as controls (solid lines, triangles). The SDS concentration in supplemented cultures was monitored concurrently (dashed lines, circles). The values plotted are the means of three replicates; error bars represent standard deviation.

The studied strain’s inability to grow in M9 minimal medium supplemented with alkyl sulfates as the sole carbon and energy sources suggested that this class of compounds may have a strong inhibitory effect on *Psychrobacter* sp. The estimation of the minimal inhibitory concentration of SDS for strains DAB_AL62B and DAB_AL43B was as low as 0.01% (*w/v*, 0.35 mM), far below the values typical for SDS-decomposing strains (which could reach 1% or higher) [26]. To examine whether the presence of the SLF module is of adaptive value for the pP62BP1 host strain, we examined the growth dynamics of the two strains in LB medium supplemented with SDS to the final concentration of 0.01% for 120 h. Simultaneously, the change in SDS concentration in the culture supernatant was monitored using the Stains-All dye method (Figure 2). In the case of DAB_AL62B, the observed decomposition of SDS correlated with the increase in the OD_600_ of the culture. In contrast, strain DAB_AL43B was not able to overcome the deleterious effect of SDS.

To confirm that the presence of the *slfRCHSL* gene cluster alone is sufficient to enable the host cell to decompose alkyl sulfates, we cloned the SLF module in the vector pABW1, which is capable of replication in *Escherichia coli* DH5α. Two plasmids were obtained: pSLF1, containing the whole module (*slfRCHSL*), and pSLF2, lacking the *slfR* gene. In both cases, the obtained derivatives became capable of SDS decomposition (Figure S6a).

### 2.3. The slfCHSL Gene Cluster Constitutes an Operon

**Figure 3:**
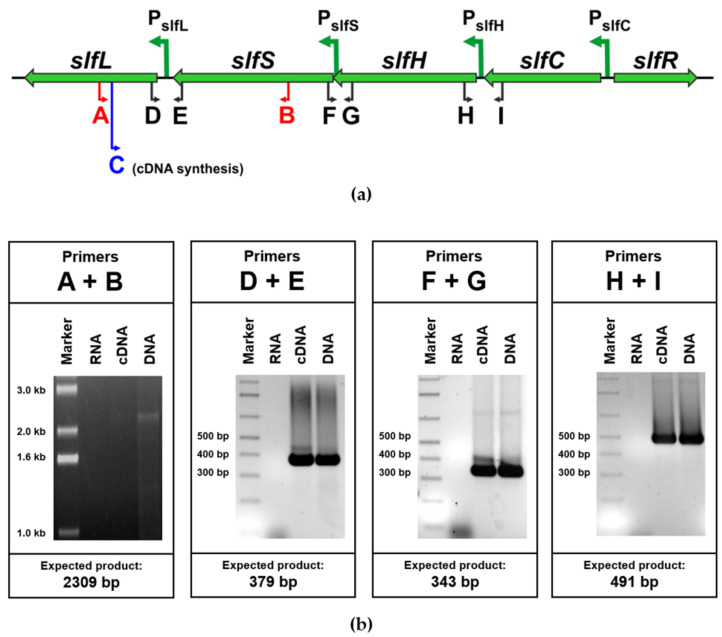
(**a**) Transcriptional organization of the SLF module. The orientation of the predicted promoter sequences is marked by green arrows. The letters A–I indicate binding sites for the primers used in the RT-PCR analysis, including the primers used for a negative control reaction (A + B, in red) and the primer used for cDNA synthesis (C, in blue). (**b**) Agarose gel electrophoresis of RT-PCR products. The analysis was performed using total RNA isolated from *E. coli* DH5α (pSLF1) grown in LB supplemented with SDS. The sizes of the amplified fragments were confirmed by comparison with the marker lane. Control reactions were performed with Dnase-treated RNA and pSLF1 DNA templates (negative and positive controls, respectively).

To examine whether the *slfC*, *slfH*, *slfS*, and *slfL* genes may be co-transcribed, a set of specific primers was designed for a reverse-transcription PCR analysis (Figure 3a). The cDNA obtained from an *E. coli* DH5α derivative carrying the pSLF1 plasmid was used as a template. The total RNA of the strain was isolated from a culture grown on LB medium supplemented with SDS to the final concentration of 0.01%. An RT-PCR was performed with (i) primers D and E, which were specific to a region that spans the *slfL* and *slfS* genes; (ii) primers F and G, spanning the *slfS* and *slfH* genes; and (iii) primers H and I, spanning the *slfH* and *slfI* genes. In all cases, the amplification of fragments of the expected sizes was achieved (Figure 3b). Each of the primer pairs used in the RT-PCR was also used in standard PCR reactions with pSLF1 DNA (positive control) and RNA isolated from *E. coli* DH5α (pSLF1) grown in the presence of SDS and treated with DNase (negative control). PCR products with the expected sizes were obtained exclusively in the former case, which confirmed the specificity of the amplification reactions. Additionally, a negative control reaction with primers A and B, spanning the region that encompasses the target site of the primer used for the cDNA synthesis (C), resulted in a product only when pSLF1 DNA was used as a template, as expected (Figure 3b).

In summary, the RT-PCR experiment demonstrated that the *slfCHSL* gene cluster constitutes an operon. Further investigation was aimed at the identification of potential internal promoters and determining how the main promoter of the operon, P_slfC_, is regulated.

### 2.4. Expression of the slfCHSL Operon Is Repressed by SlfR and Induced by Lauryl Aldehyde

**Figure 4:**
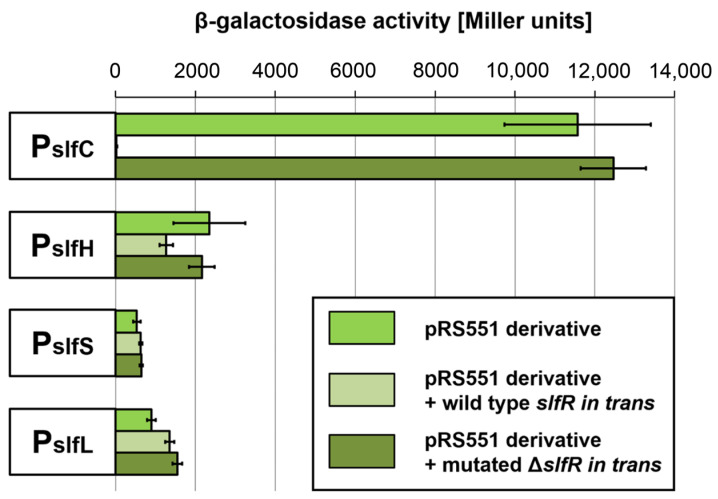
Identification of promoters within the *slfCHSL* gene cluster and the influence of the expression of the *slfR* gene in trans on their activity. The graph presents the β-galactosidase activity in *E. coli* MC1000 carrying (i) the derivatives of the promoter probe vector pRS551 for *lacZ* transcriptional fusion with the P_slfC_, P_slfH_, P_slfS_, and P_slfL_ promoters, and (ii) the same plasmids in the presence in trans of plasmid pCF-*slfR* (induction of *slfR* expression) or pCF-Δ*slfR* (induction of mutated *slfR* expression). The values plotted are means of 8 replicates; error bars indicate standard deviation.

In order to analyze the promoter(s) located within the SLF module, DNA sequences upstream of the *slfC*, *slfH*, *slfS*, and *slfL* genes were separately amplified by PCR and inserted into the promoter probe vector pRS551 to generate transcriptional fusions with a promoterless *lacZ* reporter gene. The resulting constructs were introduced into *E. coli* MC1000, and β-galactosidase activity assays were used to quantify the strength of putative promoters (Figure 4). The results indicated the presence of (i) a strong promoter upstream of the *slfC* gene (P_slfC_), the main promoter of the identified operon, as well as (ii) three relatively weak promoters upstream of the genes *slfH, slfS,* and *slfL* (P_slfH_, P_slfS_, and P_slfL_).

Experiments with strains *E. coli* DH5α (pSLF1), carrying the complete SDS module and *E. coli* DH5α (pSLF2), carrying only the *slfCHSL* operon, indicated that in the absence of the *slfR* gene the SDS degradation proceeds at a higher rate (Figure S6b). This suggested that the putative transcriptional regulator SlfR may act as a repressor of the P_slfC_ promoter. In order to test this hypothesis, the *slfR* gene was cloned into the expression vector pCF430 under the control of the arabinose-inducible promoter P_BAD_. The resulting plasmid pCF-*slfR* was then introduced in trans into the *E. coli* MC1000 cells carrying the pRS551 derivatives described above. Control experiments were performed with the use of plasmid pCF-∆*slfR*, carrying a large in-frame deletion in the proximal part of *slfR* (corresponding to the 25-144 aa positions within the protein product). This was expected to result in the lack of expression of a functional regulator. The results of the promoter activity measurements showed that the P_slfC_ promoter was completely repressed when the expression of the wild-type variant of *slfR* was induced by arabinose. A similar effect was not observed in the case of the mutant variant of the *slfR* gene expressed in trans (Figure 4).

We then proceeded to identify the inducer of the expression of the *slfCHSL* operon in native conditions, with the in cis expression of the *slfR* gene. The PCR-amplified fragment of pP62BP1 encompassing the *slfR* gene and the P_slfC_ sequence was cloned into a promoter probe vector pRS551, and the resulting plasmid pRS-*slfR*-P_slfC_ was introduced into *E. coli* MC1000 in order to test the activity of the promoter in β-galactosidase assays. The plasmid pRS-P_slfC_ devoid of the *slfR* gene was used as a positive control.

**Figure 5:**
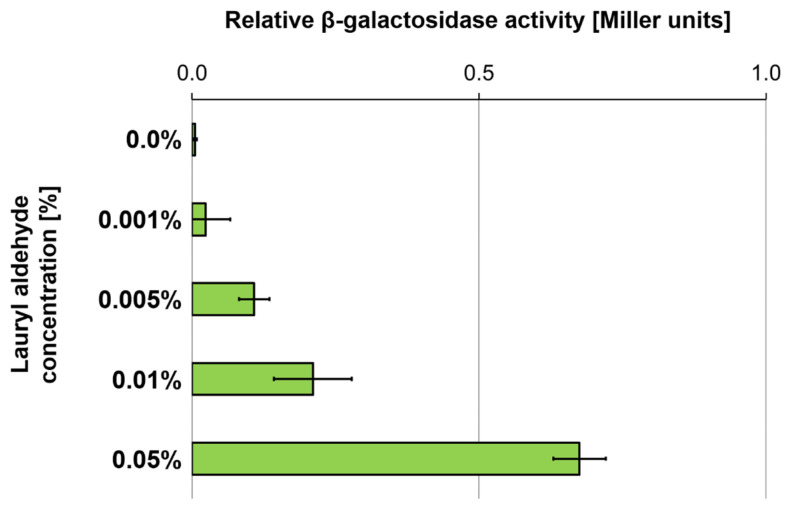
Induction of the P_slfC_ promoter by lauryl aldehyde. The graph presents the β-galactosidase activity in *E. coli* MC1000 carrying the derivative of the promoter probe vector pRS551 for *lacZ* transcriptional fusion with the P_slfC_ promoter in the presence of the *slfR* gene in cis at different concentrations of lauryl aldehyde added to the culture medium. The results are expressed as the fraction of the activity observed for *E. coli* MC1000 (pRS-P_slfC_). The values plotted are means of 8 replicates; error bars indicate standard deviation.

Based on the predicted enzymatic activity of the enzymes encoded within the *slfCHSL* gene cluster, we hypothesized that either dodecanol or lauryl aldehyde (dodecanal) might function as the inducer of the P_slfC_ promoter, as SDS hydrolysis to the former compound takes place outside of the cell [17]. Subsequently, we confirmed that the addition of lauryl aldehyde to the medium resulted in the induction of the P_slfC_ promoter, as indicated by the increase in β-galactosidase activity (Figure 5). No such effect was observed for SDS or dodecanol.

### 2.5. The SLF Module Is Flanked by Two R-M systems of the Same Specificity

Similarly to the case of the alkyl sulfatase gene, the analysis of the DAB_AL62B genome revealed that the strain’s chromosome does not contain any additional copy of either of the two R-M systems identified in pP62BP1 that flank the SLF module. No homologous system was found in either of the two sister strains, *Psychrobacter* sp. DAB_AL43B and DAB_AL32B.

**Figure 6:**
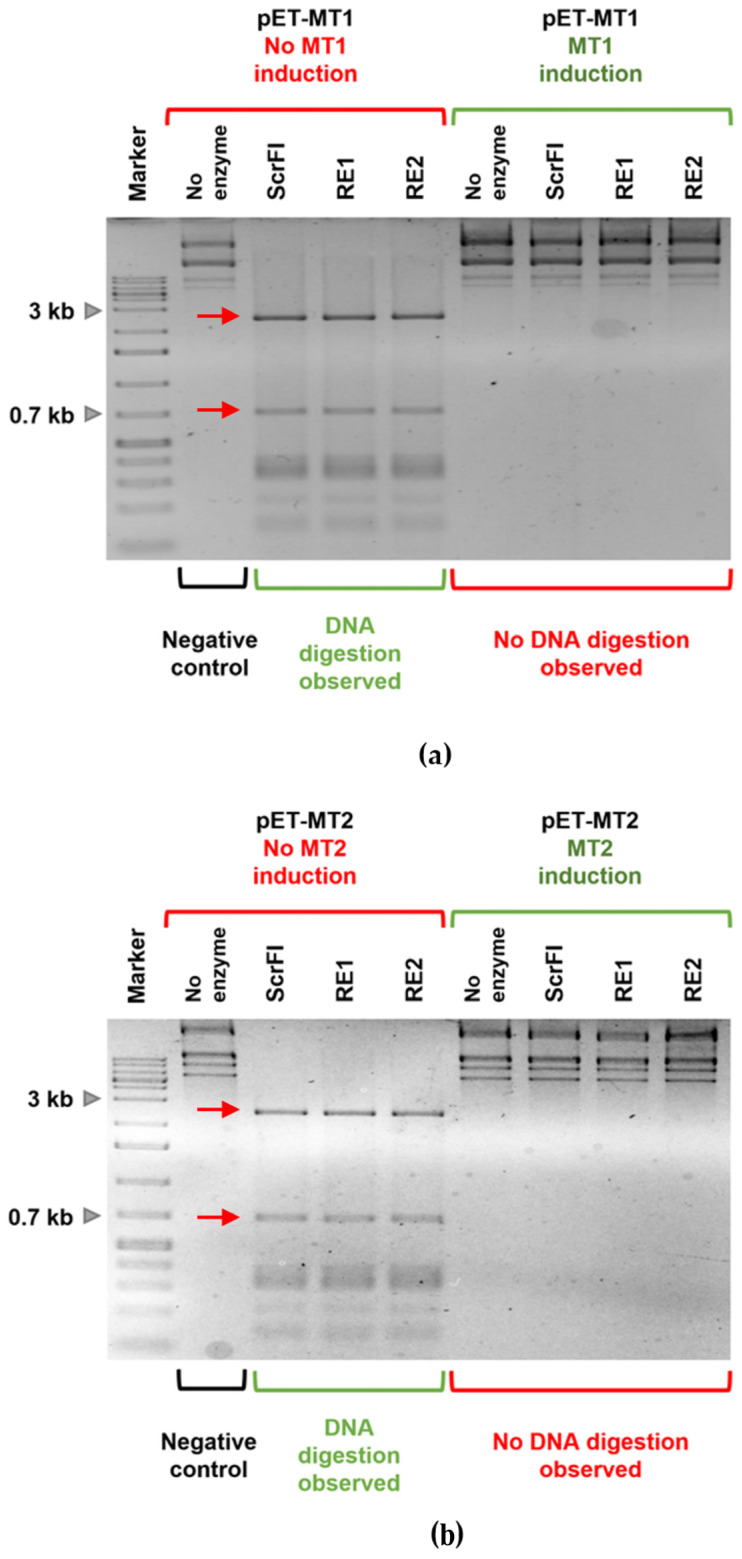
Digestion of pET-MT1 (**a**) and pET-MT2 (**b**) plasmid DNA by pP62BP1-encoded REases. The plasmid DNA was extracted from *E. coli* BL21(DE3). In both cases, digestion by the ScrFI restriction enzyme and the pP62BP1-encoded REases (RE1, RE2) was observed only in the case of the plasmid DNA isolated after the induction of MTase expression. A number of expected digestion products are visible, including the two largest fragments (2462 bp and 695 bp) indicated by red arrows.

We started the experimental analysis of the pP62BP1 R-M systems by elucidating the sequence specificity of their components. In a preliminary study, we used an in vitro expression system to obtain native REases encoded in each system, which were shown to specifically digest DNA in 5′-CCNGG-3′ sequences. For subsequent experiments, the His-tagged variants of both REases were expressed and purified using the expression system based on the pBAD/His-A vector. In parallel, derivatives of the pET28b vector carrying the genes for both MTases were obtained (pET-MT1 and pET-MT2, respectively) in order to induce the expression of their His-tagged variants. The plasmid DNA extracted from the appropriate *E. coli* BL21(DE3) strain carrying pET-MT1 or pET-MT2 was then used as a template for digestion by the purified REases (Figure 6). The commercially available restriction endonuclease ScrFI, which digests unmethylated 5′-CCNGG-3′ sequences, was used as a control. The experiment confirmed that both pP62BP1-encoded MTases are capable of modifying 5′-CCNGG-3′ sequences, thus preventing their digestion by ScrFI and both REases encoded in the studied R-M systems.

**Figure 7:**
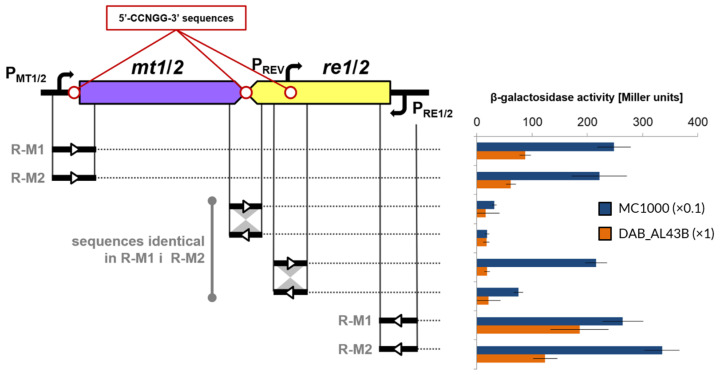
Genetic organization of each of the pP62BP1 R-M systems and the activity of putative promoter sequences in *E. coli* MC1000 and *Psychrobacter* sp. DAB_AL43B. The locations of 5′-CCNGG-3′ sites, present in each of the systems, are marked. The range and orientation of the sequences tested for promoter activity are indicated. The values plotted are means of 8 replicates; error bars indicate standard deviation. Values for the MC1000 strain were divided by 10 in order to maintain the same axis.

The genetic composition of each of the pP62BP1 R-M systems strongly suggested that the transcriptional organization of the modules might be crucial in the regulation of their activity (Figure 7). In both cases, the open reading frames encoding the MTase and the REase were oriented convergently in a ‘tail-to-tail’ manner, with overlapping STOP codons. Additionally, three 5′-CCNGG-3′ sites were identified within each system. In preliminary experiments, using β-galactosidase assays, we showed that in *E. coli* the following promoter sequences found within the pP62BP1 R-M systems were functional: (i) P_MT1_ and P_MT2_ for the MTase genes, located between a pair of imperfect inverted repeats of a 5′-CCGGG-3′ pentamer; (ii) P_RE1_ and P_RE2_ for the REase genes; and (iii) P_REV_ promoters identified next to a 5′-CCAGG-3′ sequence within each REase gene in an reverse orientation with regard to the P_RE_ promoters (Figure 7). While there were considerable differences between the nucleotide sequences encompassing the P_MT1_, P_MT2_, P_RE1_, and P_RE2_ promoters, the P_REV_ sequences were identical in the case of both systems. However, given that some species-specific factors could be involved in the transcriptional regulation of the studied R-M systems, in the next step we sought to confirm the observed promoter activity in our model *Psychrobacter* sp. strain DAB_AL43B. For this reason, the PCR-amplified fragments of pP62BP1 encompassing the putative promoter-containing fragments of R-M1 and R-M2 were cloned into a newly obtained promoter probe vector pRSPsy which could be used in both *E. coli* and *Psychrobacter* spp. (NB: *Psychrobacter* spp. naturally lack any β-galactosidase activity). In DAB_AL43B, the analysis confirmed the presence of functional promoters for MTase- and REase-encoding genes. However, the putative P_REV_ promoter was only found to provide a low level of *lacZ* transcription in this host, as with other intragenic sequences (Figure 7).

**Figure 8:**
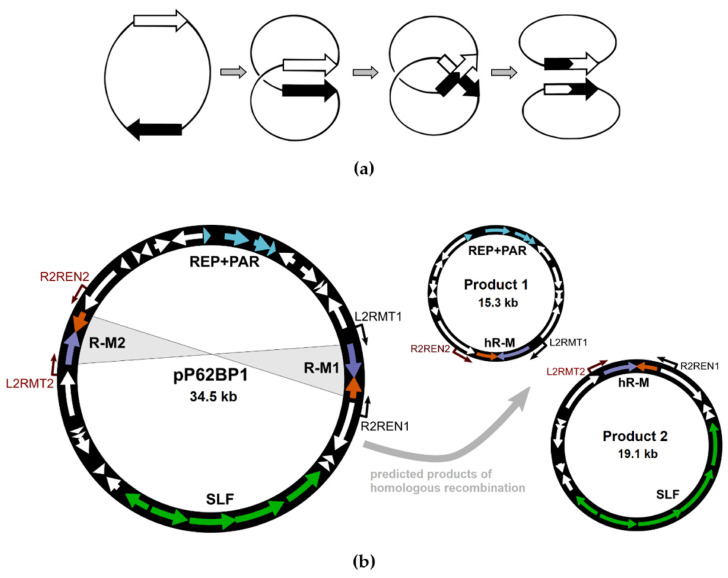
Hypothetical homologous recombination between the pP62BP1 R-M systems. (**a**) Theoretical model of DNA recombination between homologous sequences found with the same orientation in a circular DNA molecule. (**b**) Predicted products of the homologous recombination between the pP62BP1 R-M systems. Hypothetical ‘hybrid’ R-M systems (hR-M) are indicated. Neither of the predicted products was identified experimentally. Binding sites for primers used for PCR-based screening are indicated (cf. Figure S7). Colors correspond to the main genetic modules of the plasmid.

Given the high level of identity among the nucleotide sequences of the two R-M modules and their orientation within the plasmid, we hypothesized that a homologous recombination between them may result in the emergence of a *slfRCHSL*-less plasmid, still capable of replication due to the presence of the REP module (Figure 8). An emerging ‘hybrid’ R-M system would contain the coding sequences for MTase and REase under the control of the promoters P_MT1_ and P_RE2_, respectively. However, we failed to identify such a recombination event by means of a PCR-based screening with the appropriate pairs of primers (Figure S7).

## 3. Discussion

In this study, we performed a functional analysis of two types of plasmid-borne genetic modules identified in an Arctic strain of *Psychrobacter* sp.: a catabolic operon, SLF, and a pair of type II R-M systems. This served as an experimental confirmation of the in silico predictions based on comparative analyses of the putative pP62BP1-encoded genes and their products [17].

The decomposition of alkyl sulfates has been extensively studied in *Pseudomonas* spp. (e.g., [27]). Recently, the LaoABCR system for the oxidation of the products of SDS hydrolysis in *Pseudomonas aeruginosa* PAO1 was characterized [28,29,30]. In this case, both positive and negative regulation mechanisms were shown to control the studied metabolic pathway, with, i.a., SDS and long-chain acyl-CoA esters acting as inducers of gene expression in a step-wise manner [29]. It is worth noticing, however, that the genetic determinants of alkyl sulfate metabolism in *Pseudomonas* spp.—specifically, the genes for alkyl sulfatases—tend to be distributed in various parts of the genome. In contrast, the pP62BP1-borne operon represents a compact gene cluster. Further comparative studies are needed to shed light on the evolutionary history of the SLF module that gave rise to this particular genetic setup.

We hypothesize that in part it might have been shaped by the presence of the two R-M systems within the same replicon. To the best of our knowledge, a similar ‘twin’ genetic pattern has not been described to date. In this work, we showed that all the protein components of the two systems are functional. Judging by the complex transcriptional landscape of the studied genetic modules, we expect that cross-talk between the elements of the two modules maintains the two systems within a single extrachromosomal replicon. Further analysis would require a focus on the MTases encoded within the systems. They are known to act as transcriptional regulators by binding to DNA both within their target sequences and beyond [31].

Curiously, we were not able to identify products of homologous recombination between the two ‘twin’ R-M systems that would be expected given the high level of identity between their sequences. This can be explained by the presence of a putative *hicAB*-family toxin–antitoxin (TA) system in a region of pP62BP1 between the SLF module and R-M2 [17]. In this case, the formation of a plasmid devoid of the TA system caused by homologous recombination would result in the elimination of its host cell from the bacterial population. Alternatively, the hybrid R-M system predicted to emerge as the result of a recombination event may by improperly regulated and consequently act against the host genome, with the MTase failing to methylate the target sequences, thus making them prone to the cleavage by the associated REase [32]. Regardless of the mechanism, the SLF module seems to be maintained within the pP62BP1 plasmid in spite of the possibility of recombination between the R-M systems.

It is important to emphasize that the regulatory mechanism of the *slfCHSL* operon that we identified in this study has potential as an element of an inducible gene expression system that could be functional in both *E. coli* and *Psychrobacter* spp. strains. As evidenced by the results of the β-galactosidase assays, the P_slfC_ promoter could ensure a relatively high level of transcription of a gene of interest cloned through transcriptional fusion in the presence of very low concentrations of the inducer, lauryl aldehyde.

Interestingly, while performing β-galactosidase assays in search of the direct inducer of the *slfCHSL* operon using 96-well titration plates, we observed that lauryl aldehyde can apparently trigger transcription from the P_slfC_ promoter in its volatile form, acting on the bacterial cultures in non-neighboring wells. The modulation of gene expression by naturally occurring volatile organic compounds does not seem to be uncommon in microbe–microbe and plant–microbe interactions (e.g., [33,34,35])

On the other hand, lauryl aldehyde is hardly soluble in water—a trait undesirable in a potential inducer of a gene expression system. However, in an alternative design, the bacterial host of the system could be engineered to express the SlfS and SlfH proteins (cf. Figure 1a). This would enable the highly soluble SDS molecule to act as a precursor for the direct inducer of the system, facilitating the optimization of the experimental conditions for gene expression.

## 4. Materials and Methods

### 4.1. Bacterial Strains and Culture Conditions

The following bacterial strains were used in this study: *Psychrobacter* sp. DAB_AL62B [17], *Psychrobacter* sp. DAB_AL43B [13], *E. coli* DH5α (Invitrogen, Carlsbad, CA, USA), *E. coli* MC1000 [36], and *E. coli* BL21(DE3) (Invitrogen). All strains were grown in lysogeny broth (LB) or M9 minimal medium at 22 °C (*Psychrobacter* spp.) or 37 °C (*E. coli*). When necessary, the medium was supplemented with antibiotics: kanamycin (10–50 μg/mL), tetracycline (20 μg/mL), ampicillin (100 μg/mL), and rifampicin (50 μg/mL). Stock solutions (1%, *v/v*) of 1-dodecanol and lauryl aldehyde (Sigma-Aldrich, St. Louis, MO, USA) were prepared in ethanol and then diluted in the culture medium to the appropriate final concentration.

### 4.2. Genetic Manipulations

Routine DNA manipulation procedures were performed according to standard methods [37]. Plasmid DNA was introduced into *E. coli* cells by the chemical transformation method of Kushner [38] and into *Psychrobacter* sp. cells by the triparental mating procedure described previously [13]. The lists of plasmids and primers used in this study are presented in Table S4 and Table S5, respectively.

### 4.3. Alkyl Sulfate Biodegradation Assays

The biodegradation of alkyl sulfates was monitored in both solid and liquid media using Stains-All dye (Sigma-Aldrich) based on the principles described by Rusconi et al. [25]. The 1 mg/mL stock solution of Stains-All for both types of assay was prepared in isopropanol and water (50:50, *v/v*). For the substrate range assay, the tested strains were streaked on LB plates supplemented with a water solution of alkyl sulfates (0.005–0.05%, *w/v*) and the Stains-All stock solution (1%, *v/v*). The purple coloring of the medium caused by the bacterial growth was judged to be indicative of alkyl sulfate degradation.

The rate of SDS biodegradation in liquid media was determined as described by Furmanczyk et al. [26] with minor modifications. The overnight cultures of the tested *Psychrobacter* strains were diluted in fresh LB medium to OD_600_ 0.3. After 4 h, the cultures were supplemented with SDS to the final concentration of 0.01% (*w/v*). The detergent concentration in the culture along with its OD_600_ were then sampled at regular intervals (first every 4 h, then every 12 h). For the colorimetric quantification of SDS, an intermediate solution of Stains-All was prepared (1 mL Stains-All stock solution, 1 mL formamide, 18 mL deionized water). Culture samples (0.3 mL) at every time point were first centrifuged (5 min, 12,000 rpm), and then 0.1 mL of the supernatants was mixed with 0.4 mL of deionized water and 0.5 mL of the intermediate solution. After 10 min of incubation at room temperature, the absorbance of the samples was measured at 438 nm. The assay was performed in triplicate.

### 4.4. β-Galactosidase Assay

β-Galactosidase assays were performed following the method described by Schaefer et al. [39] with some modifications. Cultures of *E. coli* MC1000 or *Psychrobacter* sp. DAB_AL43B carrying derivatives of the pRS551 or pRSPsy promoter probe vectors were grown in LB medium supplemented with appropriate antibiotics on 96-well titration plates overnight at 30 °C or 21 °C with shaking. The cultures were then diluted with a fresh portion of the medium at a ratio of 1:50 (for *E. coli* MC1000 cultures) or 1:30 (for *Psychrobacter* sp. DAB_AL43B cultures) and incubated under the same conditions until reaching an OD_600_ of approximately 0.2–0.4. Assays were performed in a freshly prepared reaction mixture containing 4 mg/mL 2-nitrophenyl-β-D-galactopyranoside (ONPG), 10% PopCulture Reagent (Novagen, Madison, WI, USA), 40 mM NaH_2_PO_4_ × H_2_O, 60 mM Na_2_HPO_4_ × 7H_2_O, 10 mM KCl, 1 mM MgSO_4_ × 7H_2_O, and 0.05 M β-mercaptoethanol (pH 7). Enzymatic reactions were set up on a new 96-well plate by mixing: (i) in the case of *E. coli* MC1000, 30 μL of the bacterial culture and 170 μL of 1× concentrated reaction mixture; (ii) in the case of *Psychrobacter* sp. DAB_AL43B, 100 μL of the bacterial culture and 100 μL of 2× concentrated reaction mixture. A larger amount of bacterial culture was used in the case of strain DAB_AL43B due to the lower cell lysis efficiency of the PopCulture reagent observed for *Psychrobacter* sp. Subsequently, the measurement of the amount of product generated by ONPG hydrolysis was immediately initiated by determining the absorbance at 420 nm using a Tecan Sunrise plate reader. The value of β-galactosidase activity (in Miller units) was determined using the following formula: (1000 × (ΔA420/min))/(OD_600_ × V), where ΔA420/min is the maximum reaction rate, and V is the volume of bacterial culture used (in mL) [40]. In all assays, 8 replicates were performed. The values obtained for the same plasmid constructs in *Psychrobacter* sp. using this method are typically an order of magnitude lower than the values detected in *E. coli* MC1000.

### 4.5. Reverse-Transcription PCR Analysis

RT-PCR analysis was performed as described by Dziewit et al. [41]. Total RNA was extracted from a cell pellet of an *E. coli* DH5α (pSLF1) culture. Primer C was used for the first-strand synthesis of cDNA. Different combinations of primers A, B, D, E, F, G, H, and I were used for the PCR amplification with cDNA as the template (Table S5).

### 4.6. Protein Overexpression and Purification

The overexpression of the SlfR protein (or its mutated form) was achieved using the expression vector pCF430. The appropriate coding sequences were cloned under the transcriptional control of the tightly regulated P_BAD_ promoter. Transcription was induced by the addition of arabinose to a final concentration of 0.2% after the dilution of the overnight culture of *E. coli* MC1000, as described above (Section 4.5).

The overexpression and purification of the His-tagged REases using the pBAD/His-A system was performed as described by Dziewit et al. [41]. Optimal conditions for DNA cleavage by the recombinant REases were determined empirically (different temperatures and buffers were tested). The conditions used in this study were as follows: 1 × Buffer R (Thermo Scientific, Waltham, MA, USA; 10 mM Tris-HCl (pH 8.5 at 37 °C), 10 mM MgCl_2_, 100 mM KCl, 0.1 mg/mL BSA); 28 °C; 15 min.

To achieve the overexpression of the His-tagged MTases, the cultures of *E. coli* BL21(DE3) strains (40 mL) carrying the derivatives of pET28b(+) were grown overnight at 30 °C with shaking. The cultures were diluted in a fresh portion of the medium (1:50), and after 4 h of incubation they were divided into two flasks. Isopropyl β-D-1-thiogalactopyranoside (IPTG) was added to one of the flasks, reaching a final concentration of 0.5 mM, in order to induce MTase expression, while glucose was added to the other flask (no induction), reaching a final concentration of 0.2%. After 2 h, bacterial pellets were obtained from the cultures, and plasmid DNA was isolated.

### 4.7. Genome Sequencing, Assembly, and Annotation

The whole-genome sequencing of the *Psychrobacter* sp. DAB_AL62B genome was performed in the DNA Sequencing and Oligonucleotide Synthesis Laboratory at the Institute of Biochemistry and Biophysics, Polish Academy of Sciences (Warsaw, Poland). Short-read bacterial genome sequencing was performed using the MiSeq instrument (Illumina Inc., San Diego, CA, USA). Genomic DNA was purified using the SDS/Phenol method [42]. DNA quality control was performed by measuring the absorbance at 260/230. Template concentration was determined using a Qubit fluorimeter (Thermo Fisher Scientific, Waltham, MA, USA), and DNA integrity was analyzed by 0.8% agarose gel electrophoresis. A DNA library was constructed using an NEB Ultra II FS kit (NEB, Ipswich, MA, USA), followed by paired-end 300 basepair sequencing (targeting at least 50× genome coverage). Sequence quality metrics were assessed using FASTQC (http://www.bioinformatics.babraham.ac.uk/projects/fastqc/, accessed on 1 November 2023). Raw sequencing reads were trimmed for quality, and residual library adaptors were removed using fastp software [43]. Cleaned Illumina reads were assembled into contigs using SPAdes software (https://github.com/ablab/spades, accessed on 1 November 2023) to obtain a high-quality draft genome bacterial isolate. Illumina sequencing yielded 2,191,236 raw reads and 649,656,972 nucleotides of sequence data. After quality trimming, 2,141,472 trimmed reads and 635,069,823 nucleotides remained. The draft genome sequence was automatically annotated using the NCBI Prokaryotic Genome Annotation Pipeline [18]. To estimate the contamination and completeness of the sequence, we used the EvalG tool integrated into the PATRIC database Genome Annotation Service [21], which implements a variant of the basic CheckM algorithm [20].

### 4.8. Bioinformatics

The draft genome sequence of *Psychrobacter* sp. DAB_AL62B was uploaded to the Type Strain Genome Server (TYGS) for a whole-genome-based and 16S rRNA gene-based taxonomic analysis [22]. The basic set of genomes used for the analysis comprised 32 representative members of the genus *Psychrobacter* (Table S1). The details of the TYGS-assisted analysis are presented in the Supplementary Material. The phylogenetic trees were visualized in iTOL [44]. Similarity searches were performed using the BLAST programs [45]. AAI-profiler [23] was used to assess the established phylogeny on the basis of the deduced proteome of *Psychrobacter* sp. DAB_AL62B. The Orthologous Average Nucleotide Identity Tool, which employs the OrthoANI algorithm [46], was used to calculate ANI values between strain DAB_AL62B and selected closely related *Psychrobacter* sp. strains (DAB_AL43B, DAB_AL32B, G); *P. cryohalolentis* K5; and *P. arcticus* 273-4. Orthologous groups were assigned using eggNOG-mapper [47]. The orthologous gene clusters were identified using the OrthoVenn 2 web service tool [24] with an E-value of 1 × 10^−5^ and an inflation value of 1.5.

### 4.9. Data Availability

The draft genome of *Psychrobacter* sp. DAB_AL62B was deposited in GenBank under the accession nos. SAXC00000000, PRJNA513483, and SAMN10713547 for the Genome, Bioproject, and Biosample, respectively.

## Data Availability

Publicly available datasets were analyzed in this study. These data can be found in GenBank under the accession nos. SAXC00000000, PRJNA513483, and SAMN10713547 for the Genome, Bioproject, and Biosample, respectively.

## Supplementary Materials

The following supporting information can be downloaded at: https://www.mdpi.com/article/10.3390/ijms25010551/s1. References [48,49,50,51,52,53,54,55,56,57,58,59] are cited in the supplementary materials.
