# Supplementary material for: A Plasmid-Borne Gene Cluster Flanked by Two Restriction-Modification Systems Enables an Arctic Strain of Psychrobacter sp. to Decompose SDS
Source: Int J Mol Sci. 2023 Dec 31;25(1):551. doi: 10.3390/ijms25010551 (PMC10779009; doi:10.3390/ijms25010551)
Supplement: Supplementary file 1 [file ijms-25-00551-s001.zip › ijms-2762453-supplementary.pdf]

SUPPLEMENTARY MATERIAL

A plasmid-borne gene cluster flanked by two restriction-modification systems enables an Arctic strain of *Psychrobacter* sp. to decompose SDS

Robert Lasek, Ignacy Piszczek, Monika Krolikowski, Adrian Sówka, Dariusz Bartosik

SUPPLEMENTARY FIGURES

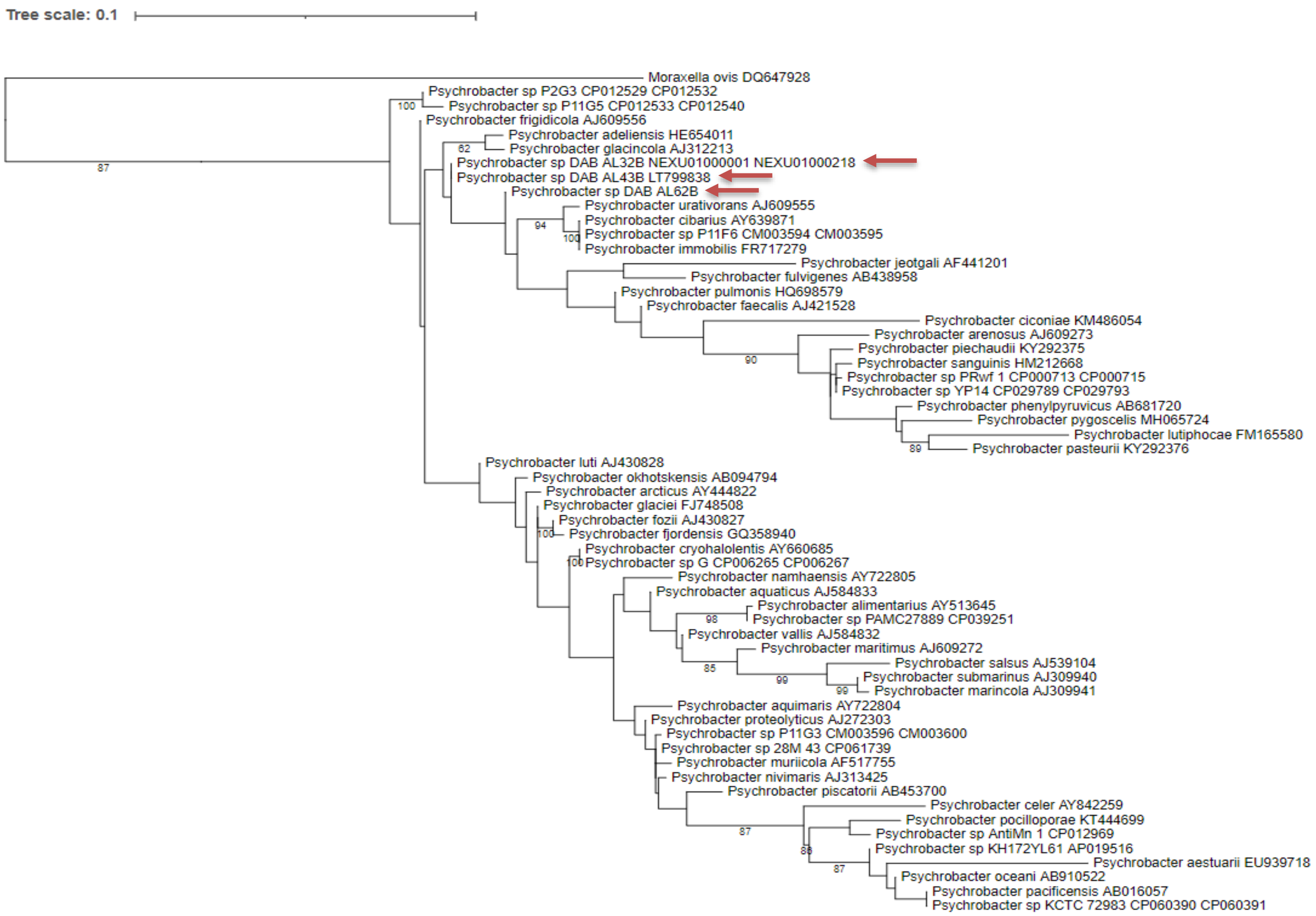

Figure S1. Maximum likelihood phylogenetic tree inferred under the GTR+CAT model from 16S rDNA sequences of representative *Psychrobacter* sp. strains and rooted by midpoint-rooting. The branches are scaled in terms of the expected number of substitutions per site. The numbers below the branches are support values >60% from ML bootstrapping. The 16S rDNA sequence of *Moraxella ovis* ATCC 33078 was used as the outgroup. The positions of strains DAB_AL62B, DAB_AL43B and DAB_AL32B are indicated by red arrows.

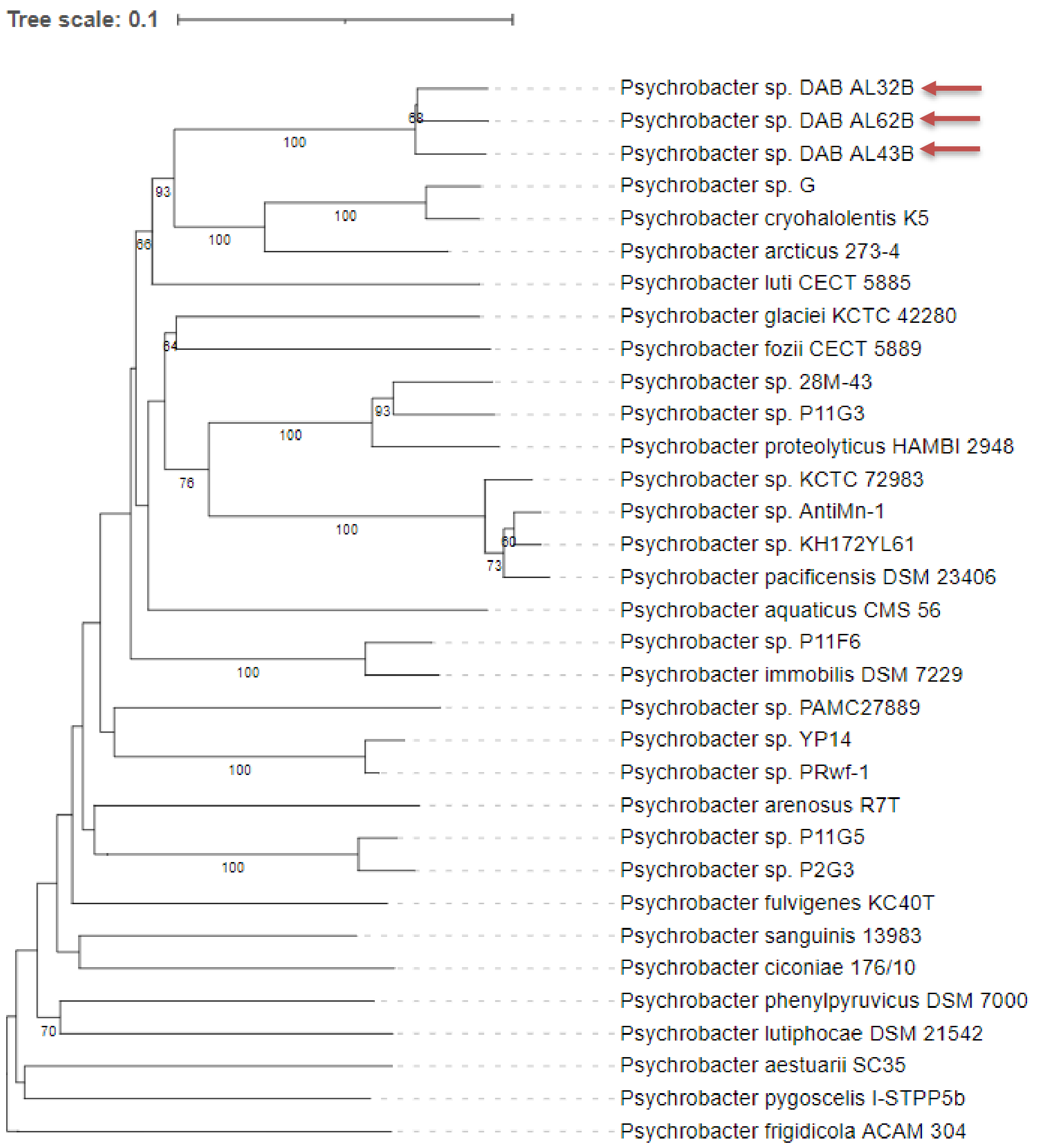

Figure S2. Phylogenomic tree inferred with FastME 2.1.6.1 [48] from GBDP distances calculated from genome sequences of representative *Psychrobacter* sp. strains. The branch lengths are scaled in terms of GBDP distance formula d5. The numbers below branches are GBDP pseudo-bootstrap support values > 60 % from 100 replications, with an average branch support of 53.0 %. The tree was rooted at the midpoint. The positions of strains DAB_AL62B, DAB_AL43B and DAB_AL32B are indicated by red arrows.

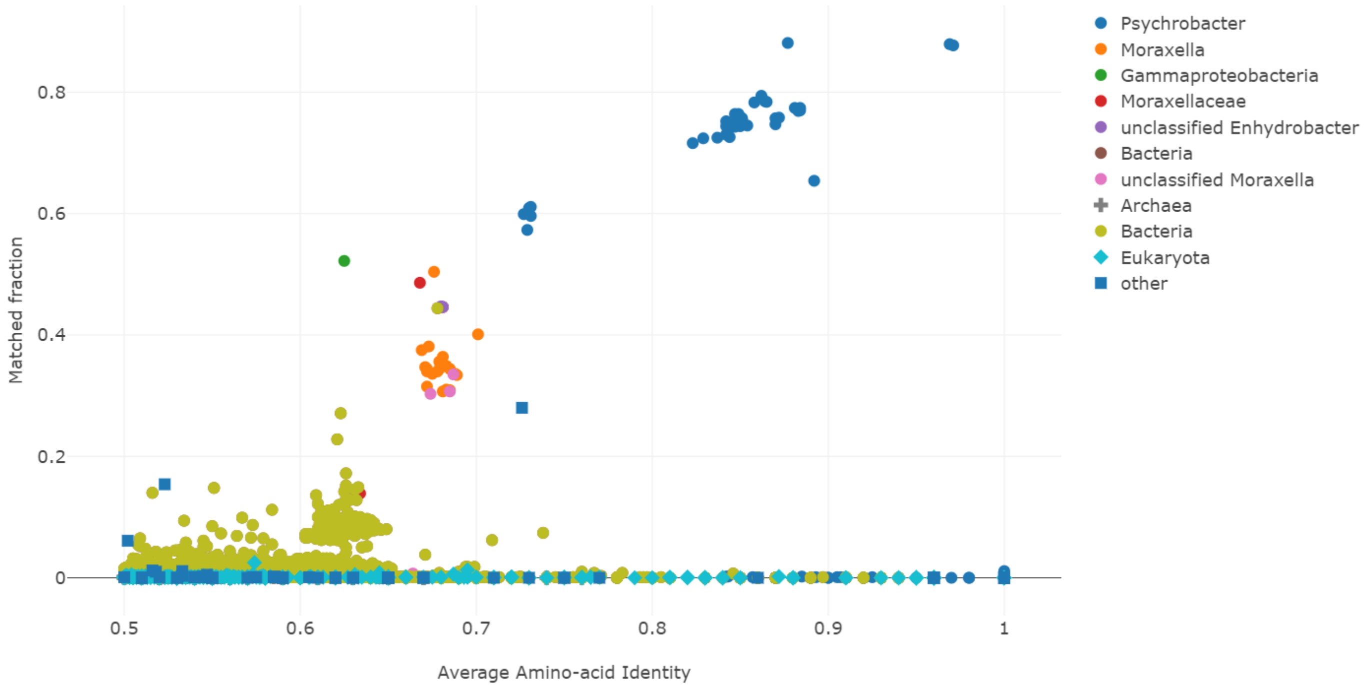

Figure S3. AAI-profiler scatterplot for *Psychrobacter* sp. DAB_AL62B. The horizontal axis shows AAI between the query and database species. The vertical axis shows the fraction of query proteins that have a match in the species. Species with higher AAI (to the right) are more closely related to the query than species with lower AAI (further to the left). Data points are coloured according to the key.

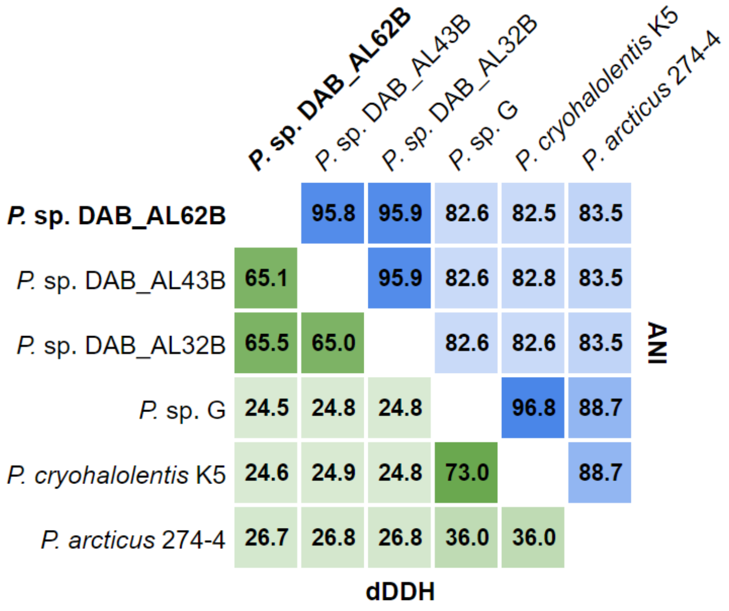

Figure S4. ANI and dDDH (d4) values for strain DAB_AL62B and closely related *Psychrobacter* sp. strains.

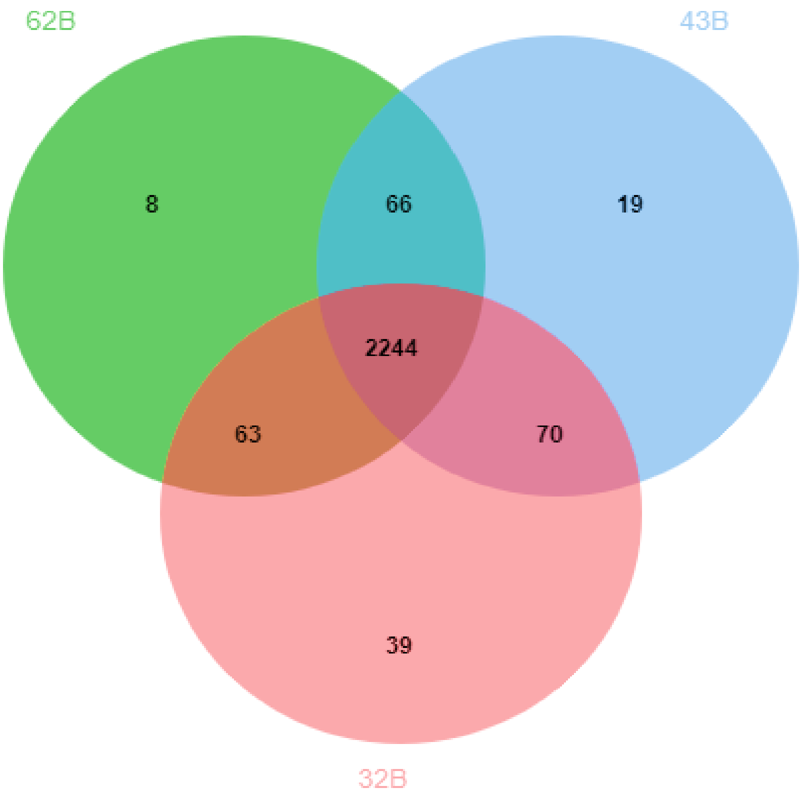

Singletons: **62B:** 172 **43B:** 328 **32B:** 233

Figure S5. Venn diagram indicating the number of shared orthologous clusters among the genomes of *Psychrobacter* sp. strains DAB_AL62B, DAB_AL43B and DAB_AL32B. The number of singletons refers to the genes for which no orthologs could be found in other species. The diagram was plotted by using OrthoVenn2 [24].

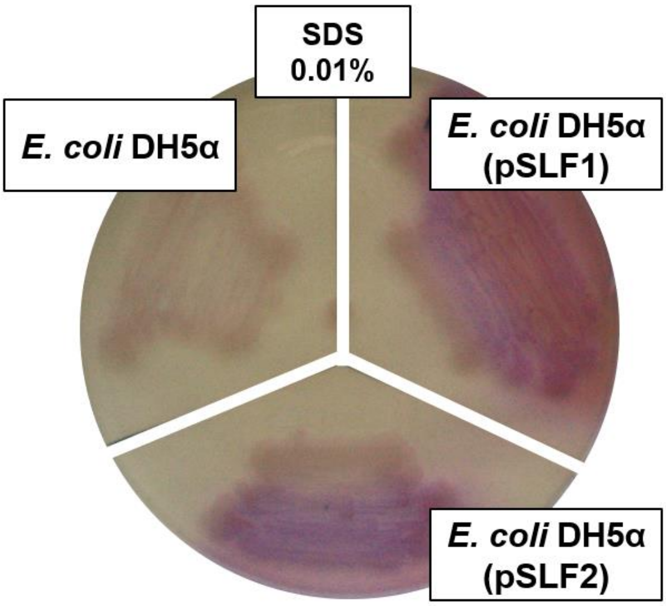

(a)

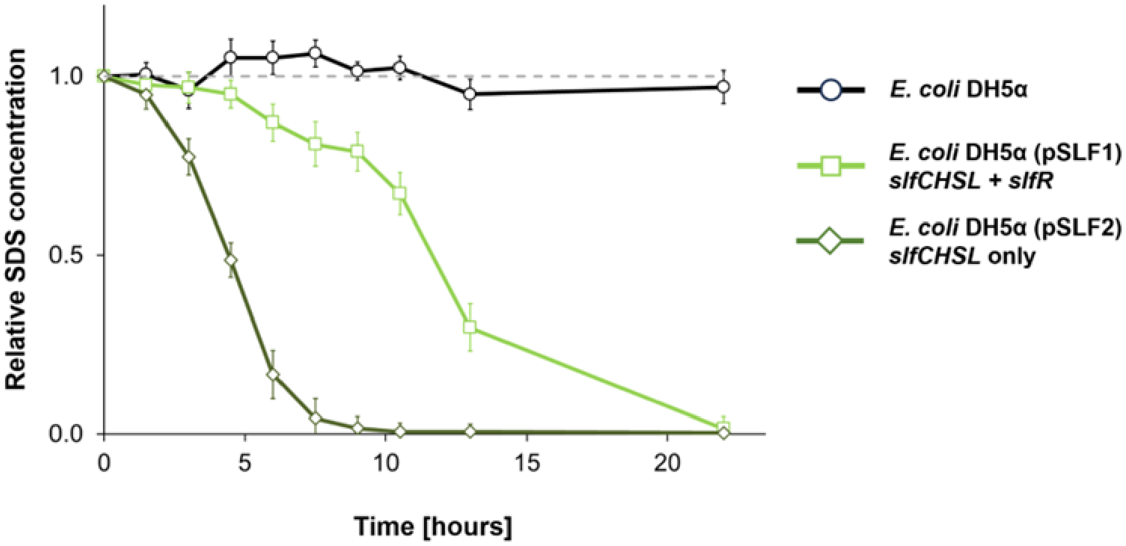

(b)

Figure S6. (a) Growth of *E. coli* DH5α and its derivatives carrying plasmids pSLF1 (*slfRCHSL*) and pSLF2 (*slfCHSL*) on LB medium supplemented with SDS to the final concentration of 0.01% (0.7 mM). **(b)** SDS degradation by *E. coli* DH5α derivatives carrying the plasmid pSLF1 (with the complete SLF module; light green line, squares) and pSLF2 (with the *slfCHSL* gene cluster only; dark green line, diamonds). *E. coli* DH5α strain served as a negative control (black line, circles). The values plotted are the means of three replicates; error bars represent standard deviation.

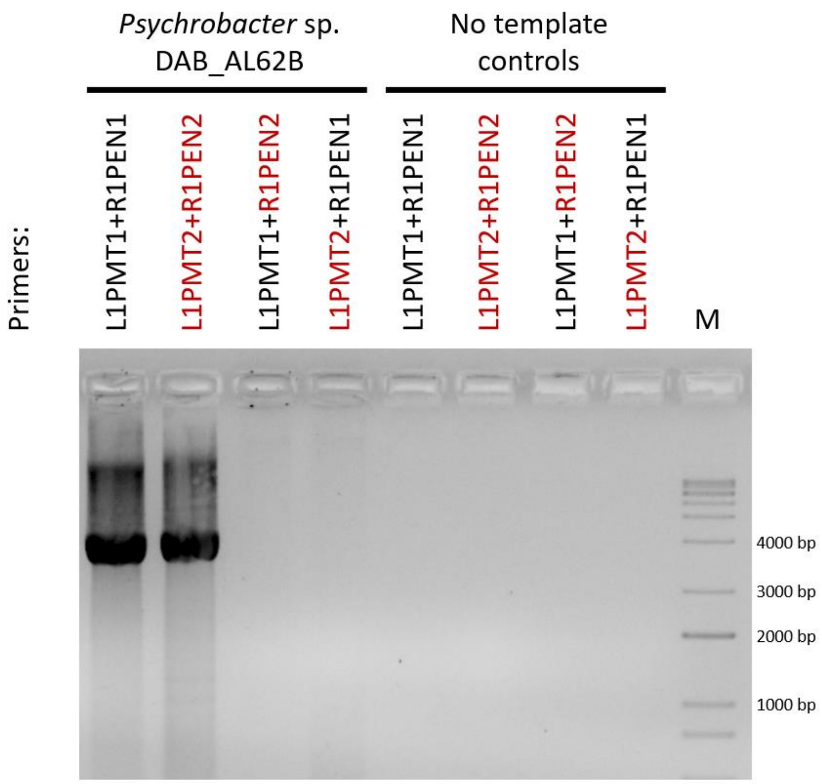

Figure S7. Representative results of the PCR-based screening aimed at the identification of a hypothetical product of homologous recombination between the pP62BP1-encoded R-M systems. Total DNA isolated from five colonies of *Psychrobacter* sp. DAB_AL62B was pooled and used as template in a set of PCR reactions performed with various primers combinations (see Figure 7 in the main text for primer binding positions). In total, 200 colonies were tested. M – DNA marker.

SUPPLEMENTARY TABLES

Table. S1. *Psychrobacter* spp. genomes used in the TYGS-assisted genome analysis of *Psychrobacter* sp. DAB_AL62B.

Strain	Base pairs	Percent G+C	No. proteins	Biosample accession
Psychrobacter sp. DAB_AL62B	3 215 484	41.97	2645	SAMN10713547
Psychrobacter sp. DAB AL43B	3 309 148	42.01	2776	LT799838
Psychrobacter sp. DAB AL32B	3 211 529	41.94	2789	NEXU01000001-NEXU01000218
Psychrobacter aestuarii SC35	2 796 792	49.64	2353	SAMEA7339708
Psychrobacter aquaticus CMS 56	3 216 408	43.1	2814	SAMN02469418
Psychrobacter arcticus 273-4	2 650 701	42.8	2120	SAMN02598544
Psychrobacter arenosus R7T	3 696 886	44.83	2930	SAMEA7339714
Psychrobacter ciconiae 176/10	2 484 493	45.74	2169	SAMEA7339718
Psychrobacter cryohalolentis K5	3 101 097	42.24	2511	SAMN02598327
Psychrobacter fozii CECT 5889	3 494 056	42.37	2873	SAMN09074959
Psychrobacter frigidicola ACAM 304	2 840 171	42.12	2299	SAMN10790460
Psychrobacter fulvigenes KC40T	3 465 773	44.09	2982	SAMEA7339726
Psychrobacter glaciei KCTC 42280	3 356 034	43.4	2780	SAMD00245646
Psychrobacter immobilis DSM 7229	3 242 871	42.84	2684	SAMN08776255
Psychrobacter luti CECT 5885	2 960 272	42.65	2463	SAMN12025092
Psychrobacter lutiphocae DSM 21542	3 172 127	41.46	2630	SAMN02441319
Psychrobacter pacificensis DSM 23406	3 060 890	43.98	2666	SAMN05660405
Psychrobacter phenylpyruvicus DSM 7000	3 099 926	41.67	2622	SAMN02743348

Psychrobacter proteolyticus HAMBI_2948	3 026 304	42.81	2460	SAMN09428662
Psychrobacter pygoscelis I-STPP5b	3 393 475	44.42	2739	SAMN10415396
Psychrobacter sanguinis 13983	3 165 790	41.27	2578	SAMEA7339774
Psychrobacter sp. 28M-43	3 115 700	42.96	2566	CP061739
Psychrobacter sp. AntiMn-1	3 138 120	43.9	2694	CP012969
Psychrobacter sp. G	3 110 043	42.42	2606	CP006265-CP006267)
Psychrobacter sp. KCTC 72983	3 081 603	43.97	2633	CP060390-CP060391
Psychrobacter sp. KH172YL61	3 188 207	43.99	4235	AP019516
Psychrobacter sp. P11F6	3 514 998	42.76	2863	CM003594-CM003595
Psychrobacter sp. P11G3	3 282 856	42.82	2649	CM003596-CM003600
Psychrobacter sp. P11G5	3 519 144	41.87	2854	CP012533-CP012540
Psychrobacter sp. P2G3	3 366 617	41.82	2741	CP012529-CP012532
Psychrobacter sp. PAMC27889	3 316 770	42.85	2740	CP039251
Psychrobacter sp. PRwf-1	2 995 049	44.84	2441	CP000713-CP000715
Psychrobacter sp. YP14	2 895 311	44.68	2345	CP029789-CP029793

Table S2. Top 10 *Psychrobacter* spp. strains showing the highest average and median amino-acid identity, and the fraction of proteins that have a match in *Psychrobacter* sp. DAB_AL62B according to AAI-profiler analysis.

**Strain**	**Average Amino-acid Identitity**	**Median Amino-acid Identity**	**Matched fraction of proteins**
Psychrobacter sp. DAB_AL43B	0.969	0.99	0.879
Psychrobacter sp. DAB_AL32B	0.971	0.99	0.877
Psychrobacter sp. JCM 18903	0.862	0.89	0.794
Psychrobacter sp. ANT_H56B	0.884	0.91	0.774
Psychrobacter cryohalolentis K5	0.883	0.91	0.772
Psychrobacter sp. ANT_WB68	0.881	0.90	0.774
Psychrobacter sp. G	0.884	0.91	0.770
Psychrobacter sp. ANT_H59	0.883	0.91	0.769
Psychrobacter sp. P11F6	0.865	0.89	0.784

Table S3. Functional distribution of *Psychrobacter* sp. DAB_AL62B proteins according to the COG classification scheme.

Predicted functions of proteins (COG categories)		Number of proteins
[C]	Energy production and conversion	177
[E]	Amino acid transport and metabolism	198
[J]	Translation, ribosomal structure and biogenesis	162
[M]	Cell wall/membrane/envelope biogenesis	136
[L]	Replication, recombination and repair	144
[P]	Inorganic ion transport and metabolism	104
[H]	Coenzyme transport and metabolism	102
[I]	Lipid transport and metabolism	113
[K]	Transcription	107
[O]	Post-translational modification, protein turnover, and chaperones	96
[T]	Signal transduction mechanisms	96
[U]	Intracellular trafficking, secretion, and vesicular transport	87
[F]	Nucleotide transport and metabolism	78
[G]	Carbohydrate transport and metabolism	70
[Q]	Secondary metabolites biosynthesis, transport, and catabolism	55
[N]	Cell motility	44
[V]	Defense mechanisms	40
[D]	Cell cycle control, cell division, chromosome partitioning	36
[S]	Function unknown	472

Table S4. Plasmids used in this work.

Plasmid	Description	Reference or source
**pABW1**	Km^r; ori pMB1; mobilizable cloning vector; *oriT* RK2	[49]
**pBAD/His-A**	Ap^r; *ori* pBR322; expression vector containing P_{BAD}	Invitrogen
**pBAD-RE1**	pBAD/His-A derivative carrying *re1* gene (amplified by PCR with primers L1PBADRE1 and R1PBADRE) inserted between NheI and EcoRI sites	This study
**pBAD-RE2**	pBAD/His-A derivative carrying *re2* gene (amplified by PCR with primers L1PBADRE2 and R1PBADRE) inserted between NheI and EcoRI sites	This study
**pBluescript SK II**	Ap^r; *ori* pMB1; cloning vector	[50]
**pCF430**	Tc^r, *ori* RK2, *oriT* RK2, P_{BAD} promoter	
**pCF-*slfR***	pCF430 derivative carrying *slfR* gene (amplified by PCR with primers L1CFSLFR and R1CFSLFR) inserted between PstI and XbaI sites	This study
**pCF-Δ*slfR***	pCF-*slfR* derivative obtained in the following steps: (1) subcloning of PstI/XbaI fragment of pCF-*slfR* in pBluescript SK II; (2) insertion of a Km^r cassette into NdeI site of the obtained product; (3) excision of the Km^r cassette and the part of *slfR* coding sequence with BamHI and autoligation of the plasmid backbone	This study
**pET28b(+)**	Km^r; *ori* pBR322; expression vector containing P_{lac}	Novagen
**pET-MT1**	pET28b(+) derivative carrying *mt1* gene (amplified by PCR with primers L1PETMT and R1PETMT) inserted between SalI and NcoI sites	This study
**pET-MT2**	pET28b(+) derivative carrying *mt2* gene (amplified by PCR with primers L1PETMT and R1PETMT) inserted between SalI and NcoI sites	This study
**pP62BP1**	natural plasmid of *Psychrobacter* sp. DAB_AL62B	[17]
**pRS551**	Ap^r; Km^r; *ori* pMB1; *lacZ* reporter gene fusion vector	[51]
**pRS-P_{slfC}**	pRS551 carrying P_{slfC} promoter (amplified by PCR with primers L1PSLFC and R1PSLFC) inserted between EcoRI and BamHI sites	This study
**pRS-P_{slfH}**	pRS551 carrying P_{slfH} promoter (amplified by PCR with primers L1PSLFH and R1PSLFH) inserted between EcoRI and BamHI sites	This study
**pRS-P_{slfL}**	pRS551 carrying P_{slfL} promoter (amplified by PCR with primers L1PSLFL and R1PSLFL) inserted between EcoRI and BamHI sites	This study
**pRS-P_{slfS}**	pRS551 carrying P_{slfS} promoter (amplified by PCR with primers L1PSLFS and R1PSLFS) inserted between EcoRI and BamHI sites	This study
**pRSPsy**	pRS551 derivative obtained in the following steps: (1) subcloning of BglII/EcoRV fragment of pP62BP1 in pABW1 between BamHI and (blunted) EcoRI sites; (2) ligation of the obtained plasmid linearized with PstI (blunted) and the PstI/SalI (blunted) fragment of pRS551.	This study
**pRSPsy-P_{MT1}**	pRSPsy carrying P_{MT1} promoter (amplified by PCR with primers L1PMT1 and R1PMT1) inserted between EcoRI and BamHI sites	This study
**pRSPsy-P_{MT2}**	pRSPsy carrying P_{MT2} promoter (amplified by PCR with primers L1PMT2 and R1PMT2) inserted between EcoRI and BamHI sites	This study
**pRSPsy-P_{RE1}**	pRSPsy carrying P_{RE1} promoter (amplified by PCR with primers L1PRE1 and R1PRE1 inserted between EcoRI and BamHI sites	This study
**pRSPsy-P_{RE2}**	pRSPsy carrying P_{RE2} promoter (amplified by PCR with primers L1PRE2 and R1PRE2) inserted between EcoRI and BamHI sites	This study

**pRSPsy-P$_{REV1F}$**	pRSPsy carrying a potential intragenic promoter sequence oter from the pP62BP1 R-M system (amplified by PCR with primers L1ARF and R1ARF) inserted between EcoRI and BamHI sites	This study
**pRSPsy-P$_{REV1R}$**	pRSPsy carrying a potential intragenic promoter sequence from the pP62BP1 R-M system (amplified by PCR with primers L1ARR and R1ARR) inserted between EcoRI and BamHI sites	This study
**pRSPsy-P$_{REV2F}$**	pRSPsy carrying a potential intragenic promoter sequence from the pP62BP1 R-M system (amplified by PCR with primers L2ARF and R2ARF) inserted between EcoRI and BamHI sites	This study
**pRSPsy-P$_{REV2R}$**	pRSPsy carrying a potential intragenic promoter sequence from the pP62BP1 R-M system (amplified by PCR with primers L2ARR and R2ARR) inserted between EcoRI and BamHI sites	This study
**pRS-*slfR*-P$_{slfC}$**	pRS551 carrying the *slfR* gene and P$_{slfL}$ promoter (amplified by PCR with primers ECOSLFR and R1PSLFS) inserted between EcoRI and BamHI sites	This study
**pSLF1**	pABW1 derivative carrying the SLF (*slfRCHSL*) module excised from pP62BP1 (with NheI and SpeI) and inserted in XbaI site	This study
**pSLF2**	pABW1 derivative carrying the *slfCHSL* gene cluster (no *slfR*) obtained by the excision of BamHI fragment from pSLF1 and autoligation of the plasmid backbone	This study

Table S5. Primers used in this work.

Primer	Sequence
**A**	ATTGTCCTTCCCGTAGTTCC
**B**	GGAGTGGAAGGCATTATCAG
**C**	TGTAGTCCCAGATGTATAAC
**D**	GGAATTCGAGACGTTAATGGCACAGG
**E**	CGGATCCTCGCCATGATAGTGTGCAG
**F**	GGAATTCTTGCGAGTGCTCGATGG
**G**	AGGATCCATCCGCTTGTGCTGCAC
**H**	GGAATTCGGCGTTGGAGCTAGTGG
**I**	TGGATCCGCGCACTGACAGAACCA
**L1ARF**	AGAATTCAGGCAGTAGCGGAGC
**L1ARR**	GGAATTCGACGGCTGTTACAACC
**L1CFSLFR**	CTCTGCAGGAGAGTAATATGGGTAGTTACATC
**L1PBADRE1**	CACATATGCAAACAAGTATTTCAAATGAAGAAGC
**L1PBADRE2**	CACATATGCAAACTACTATTTCAGATGAAG
**L1PEN1**	AGGATCCGCGCTGATTGAAGTC
**L1PEN2**	AGGATCCGCATTGCAATAGCTCC
**L1PMT1**	GGAATTCCTGCATGCCTATCCAC
**L1PSLFC**	GGAATTCCGCCACTCTGATGTAAC
**L1PSLFH**	GGAATTCGGCGTTGGAGCTAGTGG
**L1PSLFL**	GGAATTCGAGACGTTAATGGCACAGG
**L1PSLFS**	GGAATTCTTGCGAGTGCTCGATGG
**L2ARF**	AGAATTCTGGCCAAACTTCTTTGTTA
**L2ARR**	CGGAATTCCCCTGATAAAGCAAATATAG
**L2PMT2**	ATCGAATTCGGTCGGAGGATCTTGTACAATGTC
**L2RMT1**	CCTCGCGGTACAGTCAAACATATAGG
**L2RMT2**	GTTTAAGCGTATGCCGAGACACCAG
**R1CFSLFR**	CCTCTAGAGGCGCGAATGAATAGTTAAATC
**R1PBADRE**	TTGTCGACTTAAAATCCTGGGAATGGC
**R1PSLFC**	TGGATCCGGCAGTTGTGTAGTTGAC
**R1PSLFH**	TGGATCCGCGCACTGACAGAACCA
**R1PSLFL**	CGGATCCTCGCCATGATAGTGTGCAG
**R1PSLFS**	AGGATCCATCCGCTTGTGCTGCAC

**R2REN1**	CGTGTAGCTGTGGATACGCTAAACTC
**R2REN2**	GGAAGGAAGTAAGGAGTGCCTAGATG

SUPPLEMENTARY METHODS

For the phylogenomic inference, all pairwise comparisons among the genomes were conducted using GBDP and accurate intergenomic distances inferred under the algorithm 'trimming' and distance formula d5. One hundred distance replicates were calculated each. Digital DDH values and confidence intervals were calculated using the recommended settings of the GGDC 2.1 [52]. The resulting intergenomic distances were used to infer a balanced minimum evolution tree with branch support via FASTME 2.1.4 including SPR postprocessing [48]. Branch support was inferred from 100 pseudo-bootstrap replicates each. The tree was rooted at the midpoint. For an extended 16S rRNA gene analysis including sequences of not fully genome-sequenced type strains, pairwise sequence similarities were calculated using the method recommended by Meier-Kolthoff et al. [53] available via the GGDC web server [52] using the DSMZ phylogenomics pipeline [54] adapted to single genes. A multiple sequence alignment was created with MUSCLE [55]. Maximum likelihood (ML) and maximum parsimony (MP) trees were inferred from the alignment with RAxML [56] and TNT [57], respectively. For ML, rapid bootstrapping in conjunction with the autoMRE bootstopping criterion [58] and subsequent search for the best tree was used; for MP, 1000 bootstrapping replicates were used in conjunction with tree-bisection-and-reconnection branch swapping and ten random sequence addition replicates. The sequences were checked for a compositional bias using the X^2 test as implemented in PAUP* [59].
